# Endoscopic Biopsy in Gastrointestinal Neuroendocrine Neoplasms: A Retrospective Study

**DOI:** 10.1371/journal.pone.0103210

**Published:** 2014-07-28

**Authors:** Xiao Han, Yun Cui, Chuanhua Yang, Weili Sun, Jianghong Wu, Yunjie Gao, Hanbing Xue, Xiaobo Li, Lei Shen, Yanshen Peng, Hanhui Zhang, Yan Hu, Liying Zhong, Xiaoyu Chen, Zhizheng Ge

**Affiliations:** Division of Gastroenterology and Hepatology, Ren Ji Hospital, School of Medicine, Shanghai Jiao Tong University, Shanghai Institute of Digestive Disease, Shanghai, China; The Chinese University of Hong Kong, Hong Kong

## Abstract

**Background:**

Gastrointestinal neuroendocrine neoplasms (GI-NENs) are often located in the deep mucosa or submucosa, and the efficacy of endoscopic biopsy for diagnosis and treatment of GI-NENs is not fully understood.

**Objective:**

The current study analyzed GI-NENs, especially those diagnosed pathologically and resected endoscopically, and focused on the biopsy and cold biopsy forceps polypectomy (CBP) to analyze their roles in diagnosing and treating GI-NENs.

**Methods:**

Clinical data of all GI-NENs were reviewed from January 2006 to March 2012. Histopathology was used to diagnose GI-NENs, which were confirmed by immunohistochemistry.

**Results:**

67.96% GI-NENs were diagnosed pathologically by endoscopy. Only 26.21% were diagnosed pathologically by biopsies before treatment. The diagnostic rate was significantly higher in polypoid (76.47%) and submucosal lesions (68.75%), than in ulcerative lesions (12.00%). However, biopsies were only taken in 56.31% patients, including 51.52% of polypoid lesions, 35.56% of submucosal lesions and 100.00% of ulcerative lesions. Endoscopic resection removed 61.76% of GI-NENs, including six by CBP, 14 by snare polypectomy with electrocauterization, 28 by endoscopic mucosal resection (EMR) and 15 by endoscopic submucosal dissection (ESD). 51.52% polypoid GI-NENs had infiltrated the submucosa under microscopic examination. CBP had a significantly higher rate of remnant (33.33%) than snare polypectomy with electrocauterization, EMR and ESD (all 0.00%).

**Conclusions:**

Biopsies for all polypoid and submucosal lesions will improve pre-operative diagnosis. The high rate of submucosal infiltration of polypoid GI-NENs determined that CBP was inadequate in the treatment of GI-NENs. Diminutive polypoid GI-NENs that disappeared after CBP had a high risk of remnant and should be closely followed up over the long term.

## Introduction

Gastrointestinal neuroendocrine neoplasms (GI-NENs) are rare types of disease; however the incidence and prevalence are increasing rapidly worldwide [1]. GI-NENs have a wide range of malignant potential: from benign tumor (neuroendocrine tumor, NET) to poorly differentiated carcinoma (neuroendocrine carcinoma, NEC), and surgical removal is the only effective therapy. Small, well-differentiated NETs without metastasis can be resected locally and curatively [2]. A delayed diagnosis, even for a well-differentiated NET, may result in metastasis and significantly decreased survival rates.

Endoscopic techniques for removing gastrointestinal neoplasm include polypectomy, endoscopic mucosal resection (EMR) and endoscopic submucosal dissection (ESD) etc. For the treatment of GI-NENs, EMR and ESD are effective [2], [3], while polypectomy is under investigation. Snare polypectomy achieves complete resection in the majority of cases; however, the efficacy of cold biopsy forceps polypectomy (CBP) has not been reported. CBP is commonly used for removing diminutive polyps (≤5 mm) [4], and polyps of any size are routinely biopsied for pathological diagnosis before treatment. If the diminutive polyps disappeared after biopsy, the completeness of resection would be uncertain.

In this study, we analyzed GI-NENs retrospectively, especially those diagnosed and treated endoscopically from 2006 to 2012 (since EMR was certificated in 2006 and ESD was certificated in 2009) in our medical center and summarized the characteristics of diagnosis and treatment, focusing on the biopsy and CBP.

## Materials and Methods

### Patients

The clinical data of GI-NENs from January 2006 to March 2012 in Ren Ji Hospital, School of Medicine, Shanghai Jiao Tong University were reviewed. The diagnosis of GI-NEN was based on the histopathology, confirmed by immunohistochemistry of synaptophysin and chromogranin A, combining the clinical, endoscopic and imaging findings. Patients who were pathologically diagnosed for ≥24 months with complete medical data were included. Data regarding gender, age, site, size, depth of invasion, methods of discovery, treatment, status of margin by endoscopic therapy and recurrence were collected from the medical record or follow-up.

### Parameters used for the obtainment of biopsy samples

Single-channel gastroscope (GIF-H260, Olympus, Tokyo, Japan) and single-channel colonoscope (CF-H260AL, Olympus, Tokyo, Japan) were used for the procedures of routine examination. Size of tumor and depth of invasion were determined by pathology of resection specimens, but for the lesions disappeared after biopsy, the size was evaluated by open biopsy forceps. When the forceps were fully open, the length between the two jaws was approximate 5 mm of the gastric biopsy forceps (FB-25KR-1, Olympus, Tokyo, Japan) and 7 mm of the colonic biopsy forceps (FB-24UR-1, Olympus, Tokyo, Japan). The number of “bites” for obtaining biopsy was analyzed.

### Immunohistochemical Staining for Ki67

Ki67 immunohistochemical staining was performed using a mouse monoclonal anti-human ki-67 antibody (clone MIB-1, M724029, Dako, Glostrup, Denmark, dilution 1∶130). The Ki67 index was assessed as a percentage of 500–2000 cells counted in areas of highest nuclear labeling [5]. Cells stained for ki67 were counted by the ImmunoRatio software according to the methods recommended in their website (Available from URL: http://jvsmicroscope.uta.fi/immunoratio) [6].

### Statistical Methods

Data were presented as mean±SD. Statistical differences between groups were analyzed by crosstabs. A 2-sided *P*-value<0.05 was considered statistical significance.

### Ethical Approval

This study was approved by the ethical research committee of Ren Ji Hospital. Oral consent for use of clinical records was taken from patients during follow-up. Written consent was not acquired because it is a retrospective study. All patients’ records/information were anonymized and de-identified prior to analysis.

## Results

### Endoscopic Diagnosis

**Figure 1:**
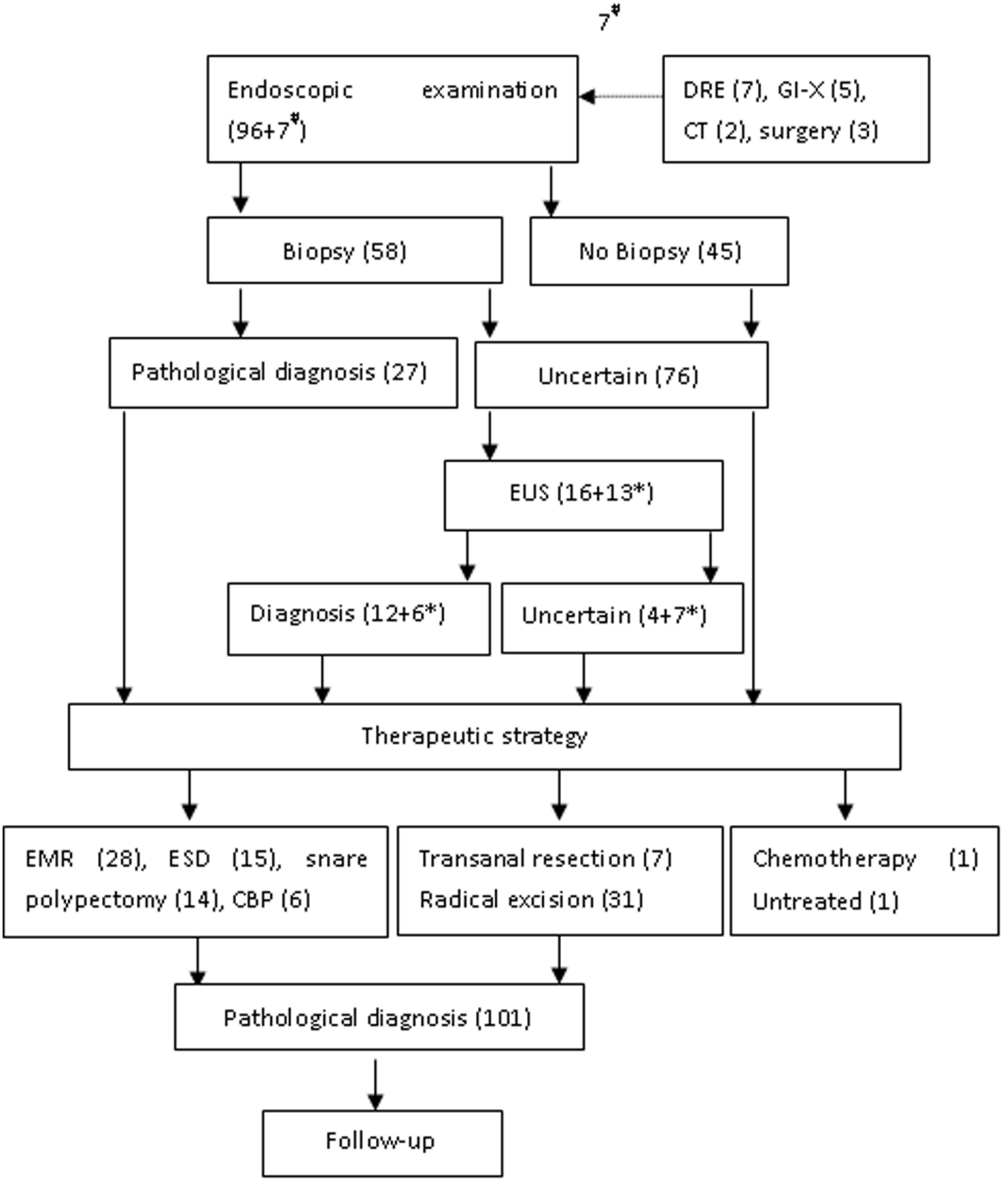
Diagnostic chart of gastrointestinal NEN. DRE: digital rectal examination; GI-X: gastrointestinal X-ray barium meal examination; CT: computed tomography; EUS: endoscopic ultrasonography; CBP: cold biopsy forceps polypectomy; EMR: endoscopic mucosal resection; ESD: endoscopic submucosal dissection; ^#^non-endoscopically discovered lesions which were further confirmed by endoscopy; *the patients examined by EUS and taken biopsy at the same time.

The procedure for diagnosis and treatment is summarized in Figure 1. One hundred and thirteen patients pathologically diagnosed as GI-NENs and treated in our medical center were reviewed, and 96 (84.96%) of them were discovered by endoscopy; seven non-endoscopically discovered lesions were further confirmed by endoscopy. Finally, the 103 patients who had received endoscopy were enrolled. The average age at diagnosis was 54.29±11.93 years (range: 29.00 to 83.00 years) and the male-to-female ratio was 53∶50 = 1.06∶1.

The endoscopic phenotypes of the 103 GI-NENs (33 gastric NENs, 68 rectal NENs, one duodenal NEN and one colonic NEN) were divided into three phenotypes (Table 1), including 33(32.04%) polypoid lesions, 45(43.69%) submucosal lesions and 25(24.27%) ulcerative lesions. Only 26.21% (27/103) patients were diagnosed pathologically based on biopsies before treatment, the diagnostic rate was significantly higher in polypoid and submucosal lesions than in ulcerative lesions (*P*<0.001); however, there was no difference in the diagnostic rate between polypoid and submucosal lesions (*P* = 0.619)(Table 2). The average “bites” for obtaining biopsy was 1.43±0.51 (range: 1 to 2), 1.90±1.29 (range: 1 to 5) and 4.36±1.34 (range: 2 to 6) for polypoid, submucosal and ulcerative lesions, respectively. The diagnostic rate, based on endoscopic ultrasonography (EUS) before treatment, was high for submucosal lesions, but was not statistically significant compared with other phenotypes (*P* = 0.395)(Table 2). Thirty-nine (37.86%) patients were diagnosed before treatment (6/18 diagnosed by EUS were confirmed by pathology of biopsies). Seventy (67.96%) patients were diagnosed pathologically by endoscopy, including 43 (41.75%) patients after endoscopic resection.

**Table 1:** The methods for diagnosis in 103 Patients.

Endoscopic Phenotype	Biopsy: No.(%)		EUS: No.(%)	
	Yes	No	Yes	No
Polypoid lesion	17(51.52)	16(48.48)	4(12.12)	29(87.88)
Submucosal lesion	16(35.56)	29(64.44)	24(53.33)	21(46.67)
Ulcerative lesion	25(100.00)	0(0.00)	1(4.00)	24(96.00)
Total	58(56.31)	45(43.69)	29(28.16)	74(71.84)

EUS: endoscopic ultrasonography.

**Table 2:** Diagnosis by biopsy or EUS for three endoscopic phenotypes.

Endoscopic Phenotype	Biopsy: No.(%)			EUS: No.(%)		
	Diagnosis	Uncertain	*P* value	Diagnosis	Uncertain	*P* value
Polypoid lesion	13(76.47)	4(23.53)	<0.001	2(50.00)	2(50.00)	0.395
Submucosal lesion	11(68.75)	5(31.25)		16(66.67)	8(33.33)	
Ulcerative lesion	3(12.00)	22(88.00)		0(0.00)	1(100.00)	
Total	27(46.55)	31(53.45)		18(62.07)	11(37.93)	

EUS: endoscopic ultrasonography.

Ki67 immunohistochemical staining was performed in 90.48% (57/63) lesions removed endoscopically. According to the WHO 2010 classification [5], 56 were G1 and one was G2 (ki67 index = 2.62%); none of them were G3.

### Treatment

Endoscopic resection removed 61.76% (63/102) of GI-NENs including six by CBP, 14 by snare polypectomy with electrocauterization, 28 by EMR and 15 by ESD; transanal excision removed seven (6.86%); radical excision removed 31 (30.39%); and chemotherapy treated one (0.98%). One patient with a rectal NEN complicated with multiple colonic ulcers was not treated at his request.

The depth of invasion in the three endoscopic phenotypes was shown in Table 3. Pathological examination showed that 77.78% (49/63) of GI-NENs resected endoscopically had infiltrated the submucosa, and 44.44% (8/18) were diminutive polypoid lesions. Twenty-six polypoid lesions were resected endoscopically; 14 provided biopsies and 12 of them were diagnosed pathologically; resection specimens without biopsies were used to diagnose12 lesions pathologically (eight lesions ≤5 mm, four lesions >5 mm and <10 mm).

**Table 3:** Depth of invasion for three endoscopic phenotypes.

Endoscopic Phenotype	Depth of invasion: No. (%)			
	Mucosa	Submucosa	Muscularis	Serosa
Polypoid lesion	13(39.39)	17(51.52)	2(6.06)	1(3.03)
Submucosal lesion	4(8.89)	39(86.67)	1(2.22)	1(2.22)
Ulcerative lesion	1(4.00)	1(4.00)	1(4.00)	22(88.00)
Total	18(17.48)	57(55.34)	4(3.88)	24(23.30)

### Follow-up

33.33% (2/6) of polypoid NETs resected by CBP had remnants in the submucosa at 4 and 2 months of follow-up, which were completely removed by ESD and transanal excision, respectively: the patients were free from disease after 34 and 19 months of follow-up. The other four patients were followed up endoscopically for 29.25 months without relapse.

Thirteen rectal NETs removed by snare polypectomy with electrocauterization, EMR and ESD were suspected of remnant by pathological examination of the positive margin or basement. According to patients’ wishes, three of them were treated by additional radical excision without any remnant examined under microscopy. Eight of the remaining 10 patients were followed up endoscopically for 44.75 months (range: 25 to 84 months) without recurrence; one patient did not receive any examination because lack of symptoms and one patient was lost to follow-up.

The 36 GI-NENs completely resected by snare polypectomy with electrocauterization, EMR and ESD were followed endoscopically for 29.22 months (six patients ≤12 months), and none of them recurred; five patients did not receive any examinations for lack of symptoms and considered themselves cured; one died of heart failure; and two were lost to follow-up.

Therefore, CBP had a significantly higher rate of remnant than snare polypectomy with electrocauterization, EMR and ESD (*P* = 0.011) (Table 4).

**Table 4:** Remnant rate of CBP and the other methods of endoscopic resection.

Endoscopy resection	Remnant: No. (%)		
	Yes	No	*P* value
CBP	2(33.33)	4(66.67)	0.011
Snare polypectomy withelectrocauterization, EMR, ESD	0(0.00)	47(100.00)	

CBP: cold biopsy forceps polypectomy; EMR: endoscopic mucosal resection; ESD: endoscopic submucosal dissection.

### Details of Gastric NENs and Rectal NENs

Only seven (21.21%) gastric lesions were diagnosed pathologically based on endoscopic biopsies (Table 5), the average “bites” for obtaining biopsy was 3.30±1.84 (range: 1 to 6); three (9.10%) were diagnosed pathologically after endoscopic resection. None were diagnosed by EUS. Twenty-three (69.70%) gastric NENs were ulcerative lesions and 12 (52.17%) of them were pre-operatively considered as adenocarcinoma, 2 of them were pathologically diagnosed NENs based on biopsies, one was considered as squamous cell carcinomas, and 3 of them were discovered malignant cells under microscopic examination. Eight (24.24%) were removed endoscopically, 24 (72.73%) were removed surgically and one (3.03%) was treated with chemotherapy.

**Table 5:** Diagnosis by biopsy for gastric and rectal NENs.

Endoscopic Phenotype	Gastric NEN: No.(%)			Rectal NEN: No.(%)		
	Diagnosis	Uncertain	*P* value	Diagnosis	Uncertain	*P* value
Polypoid lesion	4(66.67)	2(33.33)	0.009	9(81.82)	2(18.18)	0.567
Submucosal lesion	1(25.00)	3(75.00)		10(83.33)	2(16.67)	
Ulcerative lesion	2(8.70)	21(91.30)		1(50.00)	1(50.00)	
Total	7(21.21)	26(78.79)		20(80.00)	5(20.00)	

NEN: neuroendocrine neoplasm.

Twenty (29.41%) rectal NENs were diagnosed pathologically by endoscopic biopsies (Table 5), the average “bites” for obtaining biopsy was 1.89±1.23 (range: 1 to 5); and 48 (70.59%) were diagnosed pathologically after endoscopic resection. In total, 32 (47.06%) were diagnosed before treatment (6/18 diagnosed by EUS were confirmed by pathology of biopsies). Fifty-five (82.09%) rectal NENs were resected endoscopically, and 12 (17.91%) were removed surgically: seven by transanal surgery and five by radical excision. One patient was not treated at his request.

## Discussion

In our study, pathological examination of biopsy and EUS were the two major methods of pre-operative diagnosis. However, only 37.86% of lesions were diagnosed before treatment, including 21.21% gastric NENs and 47.06% rectal NENs. The causes of low pre-operative diagnosis, especially for gastric NENs, were analyzed.

Although the diagnostic rate of EUS was 66.67% for submucosal lesions, this was not statistically significant compared with polypoid or ulcerative lesions. Only 29 (28.16%) lesions were detected and 18 (17.48%) lesions were diagnosed by EUS, which suggested that EUS was complementary, especially for submucosal lesions, and that pathological examination of biopsies was the main method for diagnosing GI-NENs pre-operatively, because it was decisive diagnosis. Theoretically, GI-NENs originate from the deep portion of the epithelial glands, and biopsy would be the best method for pre-operative diagnosis. However, only 27 (26.21%) lesions were diagnosed pre-operatively and pathologically. Our data showed that 43.69% of lesions were judged as submucosal, which was higher than polypoid (32.04%) or ulcerative lesions (24.27%). Was it difficult to obtain tissues by biopsy from submucosal lesions? The rate of pathological diagnosis was significantly higher in polypoid and submucosal lesions compared with ulcerative lesion, and there was no statistical difference of the diagnostic rate between polypoid and submucosal lesions. Further analysis showed a high diagnostic rate in polypoid lesions of both gastric (66.67%) and rectal NENs (81.82%); a low diagnostic rate in ulcerative lesions of both gastric (8.70%) and rectal (50.00%) NENs; and in submucosal lesions, a high diagnostic rate of rectal NENs (83.33%) and a low rate of gastric NENs (25.00%). However, the average “bites” was more for ulcerative lesions than polypoid or submucosal lesions; and the average “bites” was more in gastric NENs than that in rectal NENs. Among 23 gastric ulcerative lesions, 18 were considered as carcinomas based on biopsies, we think these biopsies (78.26%) were effective. The possible reasons for the low diagnosis rates were: (1) the prevalence of gastric NENs is much lower than gastric adenocarcinoma in China and 52.17% of gastric NENs were considered as adenocarcinoma, especially in ulcerative lesions; and (2) the pathologists could not differentiate NENs from adenocarcinoma based on biopsies because of small specimen sizes or “crush” artifacts [7].

Only 56.31% patients provided biopsies, including 51.52% of polypoid lesions, 35.56% of submucosal lesions, and 100.00% of ulcerative lesions. The possible explanations are: (1) ulcerative lesions are always regarded as malignant tumors; (2) gastroenterologists believe that obtaining tissues by biopsy from submucosal lesions is difficult because of their location in the deep mucosa or submucosa; (3) the rate of biopsy was unexpectedly low in polypoid lesions. Considering the high pre-operative diagnostic rate for polypoid (76.47%) and submucosal lesions (68.75%), obtaining a biopsy from every lesion is strongly suggested.

Both gastric (≤1.0 cm) and rectal NETs (≤1.4 cm) that infiltrate the mucosa or submucosa, G1 or G2 (ki67 index <5%), without metastasis can be safely removed endoscopically [8], [9]. In our study, 57/102 were treated by snare polypectomy with electrocauterization, EMR and ESD; 47 cases were followed up and no one relapsed during the follow-up period, even those with positive margins. Completeness of endoscopic resection become a hot topic discussed in recent years [8], [9]. Snare polypectomy, EMR and ESD were repeatedly investigated and their efficacies for removal of GI-NENs were confirmed [2], [3], [10]. Even if positive margins were detected under pathological examination after excision, remnant was rare [11], [12]. However, we did not find a report on the efficacy of CBP. In our study, six polypoid NETs disappeared after biopsy and two had remnants in the submucosa at 4 and 2 months of follow-up, which suggested that CBP was high risk. The remnant rate of CBP was significantly higher than snare polypectomy with electrocauterization, EMR and ESD. Two main reasons were considered. (1) Complete resection using CBP was difficult. The rate of complete resection using CBP was 71% in Woods’ report [13]; 39% in Efthymiou’s report [14]; and 92.3% in the Jung’s study [4]. The high rate of completeness in the Jung’s study was achieved by spraying with indigo carmine solution; however, this technique is time-consuming and cannot be used in routine endoscopic examination. In our retrospective study, completeness was not evaluated. (2) Among the NETs removed by endoscopic resection, 51.52% (17/33) of polypoid NETs and 44.44% (8/18) of diminutive polypoid lesions had infiltrated the submucosa under microscopic examination. We hypothesize that the heat energy of snare polypectomy with electrocauterization, EMR and ESD penetrated deeper and effectively killed tumor cells; however, CBP could not achieve this. The high rate of submucosal infiltration of polypoid GI-NENs indicated that CBP was inadequate for the treatment of NET.

Should biopsy be recommended for diminutive polypoid lesions? 46.15% (12/26) of polypoid lesions resected endoscopically did not produce biopsies (including 8 diminutive ones), because some endoscopists were worried that the residual polypoid material could not be revealed after biopsy. They intended to completely remove the polyps by snare polypectomy with electrocauterization, EMR and ESD, and determine the requirement for additional therapy by pathological examination after excision. Our data showed this strategy was reasonable because all NETs removed by endoscopic resection were G1 and G2 (ki67 index <5%), and snare polypectomy with electrocauterization was an effective therapy. However, G3 NENs smaller than 1 cm were reported in previous studies [15], which suggests that a large-scale study is necessary to confirm this strategy. This strategy may be the reason for the low rate of biopsies in polypoid lesions mentioned above. And our results suggest that the optimal technique for removing diminutive polyps need further investigation.

For those diminutive polypoid lesions that disappeared after biopsy and were diagnosed pathologically as NETs, the salvage therapy comprised close endoscopic monitoring and endoscopic resection on remnant. In Park’s study, two patients with positive margins recurred at 12 and 20 months, respectively [9]; thus, our patients were followed up ≥24 months. A well-differentiated NET was reported to grow slowly and recurred after 16 years [16]; therefore, the GI-NETs with positive margins or using CBP should be followed up over the long term.

Its retrospective nature and relatively small number of patients, which reflected the low incidence of GI-NENs, limited our study. From our results, we concluded that biopsies for all polypoid and submucosal lesions would improve pre-operative diagnosis. The high rate of submucosal infiltration of polypoid GI-NENs determined that CBP was inadequate in the treatment of GI-NENs. Diminutive polypoid GI-NENs that disappeared after CBP had a high risk of remnant and should be closely followed up over the long term.
